# A Low-Serum Culture System for Prolonged *in Vitro* Toxicology Experiments on a Macrophage System

**DOI:** 10.3389/ftox.2021.780778

**Published:** 2021-12-06

**Authors:** Bastien Dalzon, Anaelle Torres, Julie Devcic, Daphna Fenel, Jacques-Aurélien Sergent, Thierry Rabilloud

**Affiliations:** ^1^ Chemistry and Biology of Metals, Université Grenoble Alpes, CNRS UMR5249, CEA, IRIG-DIESE-LCBM-ProMIT, Grenoble, France; ^2^ Institut de Biologie Structurale, Université Grenoble Alpes, CEA, CNRS, Grenoble, France; ^3^ Toxicological and Environmental Risk Assessment Unit, Solvay SA, Neder-Over-Heembeek, Belgium

**Keywords:** macrophages, effect persistence, silica, cobalt, pigments, immunotoxicology

## Abstract

Immunotoxicology *sensu lato* comprises not only toxicity toward immune cells, but also biological reactions from immune cells exposed to toxicants, reactions that may have deleterious effects at the organismal level. Within this wide frame, a specific case of interest is represented by the response of macrophages to particulate materials, with the epitome examples of asbestos and crystalline silica. For such toxicants that are both persistent and often encountered in an occupational setting, i.e. at low but repeated doses, there is a need for *in vitro* systems that can take into account these two parameters. Currently, most *in vitro* systems are used in an acute exposure mode, i.e., with a single dose and a readout made shortly if not immediately after exposure. We describe here how adequate changes of the culture methods applied to the murine macrophage cell line J774A.1 enable longer periods of culture (several days), which represents a first opportunity to address the persistence and dose-rate issues. To respond to this, the protocol uses a reduction in the concentration of the animal serum used for cell culture, as well as a switch from fetal to adult serum, which is less rich in proliferation factors. By doing so, we have considerably reduced cell proliferation, which is a problem with cell lines when they are supposed to represent slowly-dividing cells such as resident macrophages. We also succeeded in maintaining the differentiated functions of macrophages, such as phagocytosis or inflammatory responses, over the whole culture period. Furthermore, the presence of serum, even at low concentrations, provides excellent cell viability and keeps the presence of a protein corona on particulate materials, a feature that is known to strongly modulate their effects on cells and is lost in serum-free culture. Besides data showing the impact of these conditions on macrophages cell line cultures, illustrative examples are shown on silica- and cobalt-based pigments.

## Introduction

Macrophages are an important cell type to consider in immunotoxicology *sensu lato*, i.e., not only the situations where the functions of the immune system are decreased by a toxicant so that it does not play its full protective role any longer, but also the situations where a sustained activation of the immune system and especially the innate immune system is part of the pathological process. The epitome of this latter situation is represented by chronic inflammatory pulmonary diseases such as asbestosis and silicosis, where a sustained inflammation is observed. In the frame of the 3R principles (Refine Reduce Replace) aiming at limiting the use of laboratory animals, it is highly desirable to have *in vitro* models that mimic as closely as possible the *in vivo* situation.

In the case of macrophages, the *in vivo* situation is quite complex because of the plasticity and heterogeneity of macrophages. Indeed, two main different macrophage populations exist, i.e., the tissue resident macrophages and the monocyte-derived macrophages. It has been shown that tissue resident macrophages derive from the fetal liver (Ginhoux and Guilliams, 2016), have a prolonged lifespan in the body (Murphy et al., 2008) and are maintained by self renewal (Hashimoto et al., 2013; Ginhoux and Jung, 2014). In the peritoneal cavity, resident macrophages and monocyte-derived macrophages are phenotypically and functionally different (Ghosn et al., 2010), so that their dynamics can be studied in different conditions. Such studies have shown that monocyte-derived macrophages are responsible for acute inflammatory responses, and disappear after the acute inflammatory phase (Cassado Ados et al., 2015). The situation is however made more complex by the fact that a small proportion of the monocyte-derived macrophages can “*trans*-differentiate” into resident macrophages and replace the embryo-derived resident macrophages over time or in situations where the original embryo-derived resident macrophage population is depleted, e.g., by a macrophage-targeting infectious agent (Bain et al., 2016).

Thus in terms of immunotoxicology, it would be highly desirable to have at hand an *in vitro* model mimicking the resident macrophages, i.e. able of a prolonged lifespan *in vitro*, with a slow proliferation rate, and keeping the essential macrophage functions such as phagocytosis, ability to produce inflammatory response, and also the ability to reach different polarization states.

Because of the high interest in macrophages, numerous models have been described in the scientific literature. The oldest ones are both differentiated macrophages isolated *ex vivo* (e.g., thioglycollate elicited peritoneal macrophages (Adams, 1979) and cell lines of murine (RAW264.7, P388D1, J774A.1, to name just a few), rat (NR8383) and human (HL-60, THP-1, U937) origin. However, the human cell lines require to be chemically differentiated *in vitro*, most often by phorbol esters (Rovera et al., 1979; Bernal and Chen, 1982; Tsuchiya et al., 1982).

More recent macrophage systems have focused on primary cells, obtained from adult precursors, mostly bone marrow for murine cells (Schleicher and Bogdan, 2009) and monocytes for human cells (Davies and Gordon, 2004). More recently, human macrophages were prepared from pluripotent stem cells (Gutbier et al., 2020).

Unfortunately, none of these systems is completely satisfactory. Monocyte and bone-marrow derived macrophages have all the characteristics of *in vivo* monocyte-derived macrophages. As such, they do not proliferate at all and can be maintained *in vitro* for very limited time, up to only a few days after full differentiation (Aude-Garcia et al., 2016). Production of macrophages from pluripotent stem cells is rather complex, and their survival capacity once fully differentiated has not been evaluated yet. Phorbol ester-differentiated cell lines also show no proliferation capacity. Moreover, they de-differentiate over time when the phorbol ester stimulus is removed (Bertram et al., 2008). However, the phorbol ester stimulus cannot be kept permanently because of its toxicity.

Rodent cell lines present the opposite problem. While they keep excellent differentiated characteristics without the need for any chemical stimulus, they also keep a high proliferation rate. This may not be a problem for short-term studies, but it is for longer term studies. As an example, a recent study on the persistence of silver nanoparticles in macrophages has concluded to a decrease of intracellular silver content over time (Dalzon et al., 2019b). However, careful examination of the data shows that the total silver content is almost constant but that the number of cells increases during the experiment, thereby explaining the effect. Consequently, some authors have preferred to switch to serum-free culture conditions, as common for the rat NR8383 model system (Wiemann et al., 2016). However, such conditions allow for a very limited cell survival over time and are thus not suitable for any longer-term studies. Moreover, serum-free conditions are not physiological, as even alveolar macrophages bathe in pulmonary surfactant that does contain serum proteins (Wattiez et al., 2000).

Thus, there is a need for an *in vitro* system that would allow studies lasting longer than the classical overnight exposure. This would be of high interest in toxicological studies where a persistent effect has to be studied. Currently, this notion of chronicity and persisting effects is typically monitored using epidemiologic studies (Dockery et al., 1993), and *in vivo* studies (Miller et al., 1999; Oszlánczi et al., 2011; Tian et al., 2015; Boudard et al., 2019). Chronic or long-term exposures have rarely been studied *in vitro* beforehand in order to determine the cellular and molecular mechanisms behind the phenomenon, mostly because of this lack of convenient *in vitro* systems.

While mineral materials represent an obvious case where such effects persistence is interesting to study, other interesting cases can be foreseen, e.g. persistent organic toxicants such as dioxins, which are known to modulate macrophages functions through their binding to the aromatic hydrocarbon receptor (Kimura et al., 2009), or polychlorobiphenyls, which chronic inflammatory effects are currently tested *in vivo* (Petriello et al., 2018), while their short-term effects can be tested *in vitro* (Wang et al., 2019).

It is for these two reasons, i.e. the lack of *in vitro* models to determine long-term toxicity and the key role played by macrophages in the evolution of inflammation [e.g., *via* the modulation of the secretion of reactive oxygen species, nitric oxide (NO) or cytokines (Fang, 2004)] as well as in tissue homeostasis (Koh and DiPietro, 2011; Landén et al., 2016), that we seeked to implement an *in vitro* model of monocyte/macrophage cell lines enabling the detection of persisting effects, in accordance with the 3 Rs (Replace, Reduce, Refine) regulation awareness, aiming at the reduction of the use of laboratory animals.

In this study, i) thanks to functional assays (Dalzon et al., 2019a, 2020b; Torres et al., 2020b), we determined the reliability of our long-term culture model after changing the composition of culture medium, in comparison with common culture medium. The classical 10% fetal bovine serum (FBS) supplement added to DMEM supplemented was decreased (1% instead of 10%) and replaced by adult horse serum. The change in concentration and type of serum permitted to limit growth factors and thus experiments, cells were cultivated prevented cell proliferation from inducing various stresses due to a high degree of confluence and cell detachment. Indeed, cell detachment can induce artifacts because it is necessary to change culture medium every 2 days. ii) then, we determined the main functional damage caused by long-term exposure of macrophages to various mineral particles such as silica and cobalt particles.

The results showed that switching from the classical DMEM supplemented with 10% FBS to DMEM +1% horse serum improves long-term culture conditions, restricts cell proliferation and detachment. Functional assays conducted on J774A.1 macrophages without particles showed that both acute exposure (24 h to DMEM +10% FBS) and long-term exposure (10 days to DMEM +1% horse serum) provided exploitable results. Indeed, the functional capacity (phagocytosis and inflammatory) of J774A.1 cells were maintained. Thus, after 10 days, it was possible to assess the modulation of the main functions of macrophages exposed to mineral particles in comparison to controls without treatment.

## Materials and Methods

### Cell Culture and Viability Assessment

The J774A1 cell line (mouse macrophages) was purchased from European cell culture collection (Salisbury, UK). Cells were routinely propagated in DMEM supplemented with 10% fetal bovine serum (FBS) in non-adherent flasks (Cellstar). For short-term (24 h) experiments, cells were cultivated in 12-well non-adherent plate (CellStar, catalogue number 665102). For cell culture for medium-term (4 days) and long-term (10 days) experiments, cells were cultivated in 12-well adherent plates (Falcon, catalog number 353043). Starting from a growing culture made in classical culture medium (supplement with 10% fetal bovine serum), the cells were seeded at 700,000 cell/ml in DMEM +1% horse serum (1% HS) in 12-well adherent plates. For the establishment of the system, control cultures were seeded in DMEM+10% FBS. The medium was then changed every 2 days for up to 10 days in culture, without any further splitting of the cells.

For *in vitro* toxicological testing, after 2 days of cell adaptation to the low serum medium (DMEM+ 1% horse serum), the medium was changed and the cells exposed to the test substance. After the exposure period (usually 24 h), the medium was removed and replaced by fresh DMEM+1%HS medium, which was changed every 2 days during the recovery period (respectively 3 or 9 days). The same medium changes were applied to control (untreated) cultures. To obtain reference values of the effects of the substances tested, acute exposures, i.e., 24 h exposures with readouts made immediately after exposure, were also performed. In order to take cell ageing into account, these acute exposures in the long-term experiments (4 and 10 days) were carried out on days 3 and 9, respectively. Cell viability was measured using FACScalibur flow cytometer equipped with CellQuest software program (6.0, Becton Dickinson Biosciences, Le Pont-de-Claix, France). Cells were previously dyed with propidium iodide (480 nm excitation and 600 nm emission) at 1 μg/ml final concentration. For every test substance, LD20 was assessed in acute exposure mode in standard short-term (24 h) experiments.

**FIGURE 1:**
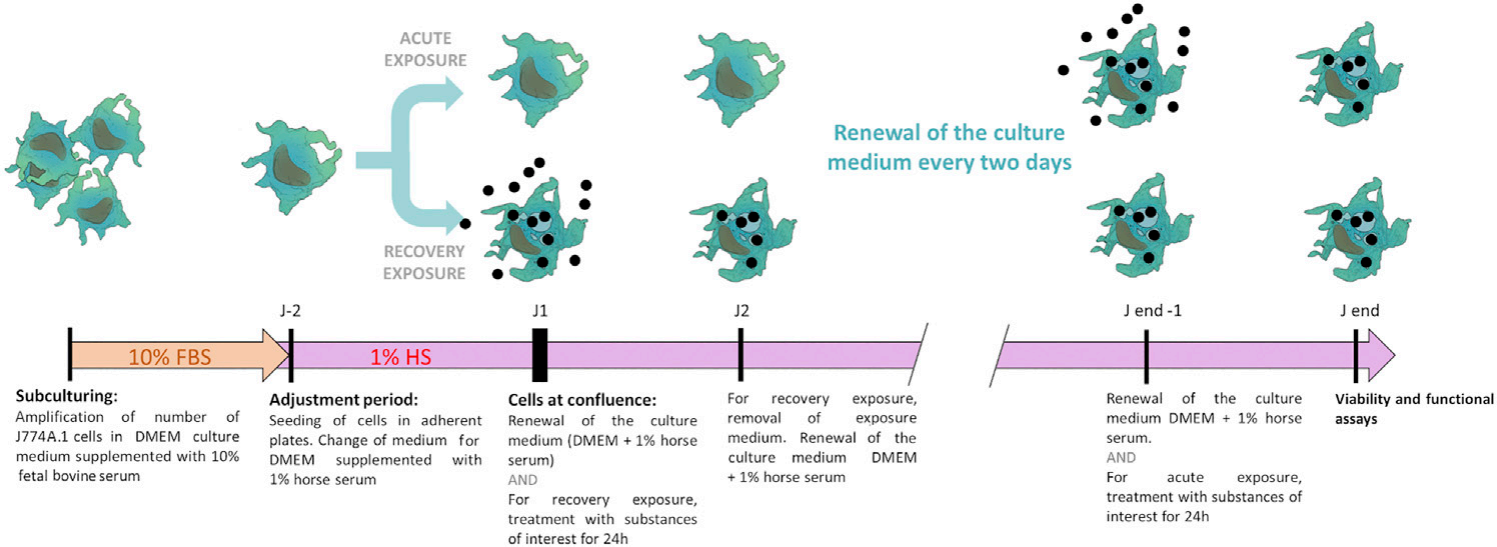
Schematic representation of the two-phase culture system. The cells are first amplified in enriched culture medium (containing 10% fetal bovine serum), then transferred in the low-serum culture medium (containing 1% adult horse serum), to which they are first adapted. The exposure and the recovery phases are then carried out, prior to final biological endpoints readout.

Overall, the culture and exposure system is schematized in Figure 1.

### Crystal Violet Quantification

The cell layer was rinsed with PBS and fixed *via* 1 ml of mix of ethanol 50% v/v and acetic acid 1% v/v (an acid alcohol solution) for 30 min. The cells were stained by crystal violet with a concentration of 4 μg/ml (final concentration) in PBS at room temperature for 30 min. Then, cells were rinsed with PBS before elution with 1 ml of acid alcohol solution. The released color was read at 590 nm using a Jenway 7,315 spectrophotometer. To convert the crystal violet measures into cell numbers, a calibration curve was constructed by seeding a known number of cells on adherent plates for 18 h, so that the cells could attach but did not have time to proliferate. These cell layers were then submitted to the crystal violet protocol, and the absorbance values used to build the calibration curve.

### Particle Characterization

Cobalt aluminate CoAl_2_O_4_, also named Pigment Blue 28 (PB28) was purchased from Kama Pigments (Montreal, Canada). Crystalline aluminum oxide spinel (Corundum) was purchased from Sigma-Aldrich (ref#ERMFD066). These materials were received in powder form. Then, 100 μg/ml were suspended in gum arabic (previously sterilized at 80 °C for 24 h). The final particular suspension was sterilized for 24 h at 80°C. The suspension was sonicated with a sonicator equipped with a Cup Horn probe (Vibra cell VC 750, VWR, Fontenay sous Bois, France) with the following settings: time = 30 min–1 s ON, 1 s OFF—Amplitude = 60%, corresponding to 90 W/pulse. The sterile suspension was then used on cells.

Silica-based pigments (tiger eye, agate and jasper) were purchased from Kremer Pigmente (Aichstetten, Germany). Jasper is quartz containing iron oxide, giving it its brown color. Agate is a form of chalcedony, i.e. formed by quartz and moganite microcrystallites, colored by iron oxide. Both quartz and moganite are silica, crystallizing in different systems. Tiger eye is composed of fibrous assemblies of quartz and amphibole (iron and magnesium silicate) microcrystallites. These pigments are sized by sieving to a nominal size of 5 µm. Opposite to colbalt aluminate, silica-based pigments suspend well in water. Resuspension was carried out as previously described (Dalzon et al., 2019b; Torres et al., 2020b). The commercial powders were solubilized in water at 10 mg/ml, dispersed in a water sonication bath. The suspensions were then sterilized by pasteurization at 80°C overnight. They were used after a last sonication step of 10 min.

For characterization by transmission electron microscopy, samples were first diluted to 100 μg/ml. Then, 3.5 µl were added to a glow discharge grid coated with a carbon supporting film for 5 min. The excess solution was soaked off by a filter paper and the grid was air-dried. The images were taken under low dose conditions (< 10 e-/Å^2^) with defocus values between 1.2 and 2.5 μm on a Tecnai 12 LaB6 electron microscope at 120 kV accelerating voltage using CCD Camera Gatan Orius 1,000.

### Phagocytic Assay

Phagocytosis capacity of J774A.1 macrophages was studied by internalization of yellow/green fluorescent latex beads (Sigma-Aldrich #L4655). The percentage of phagocytic cells and their mean fluorescent intensity were measured using FACScalibur flow cytometer with CellQuest software program (6.0, Becton Dickinson Biosciences, Le Pont-de-Claix, France) as previously described in a previous publication (Torres et al., 2020b).

### NO Secretion

After exposure of cells to the test substance (acute or recovery exposure), cells were activated (or not) by lipopolysaccharide (LPS) (100 ng/ml final concentration) in a medium supplemented with arginine monohydrochloride (5 mM final concentration) in order to provide unlimiting substrate for the NO synthase. When LPS activation was used, LPS was added to the cells for the last 18 h in culture. The concentration of NO released in the culture medium was measured as previously described (Torres et al., 2020b), using the Griess reagent for nitrite.

### Cytokine Release

Tumor necrosis factor (TNFα) and interleukin 6 (IL-6), were measured in the supernatant from cultured cells exposed to pigments and/or activated by LPS 100 ng/ml, using the Mouse Inflammation Cytometric Bead Array kit (BD cytometric bead array, BD Biosciences, Rungis, France) according to the manufacturer’s instructions. Measurements were performed on a FACScalibur flow cytometer, and the concentrations of cytokines secreted were assessed using FCAP Array Software.

## Results

### Assessment of Culture Conditions

#### Comparison of Culture Conditions: Cell Proliferation and Cell Detachment

**FIGURE 2:**
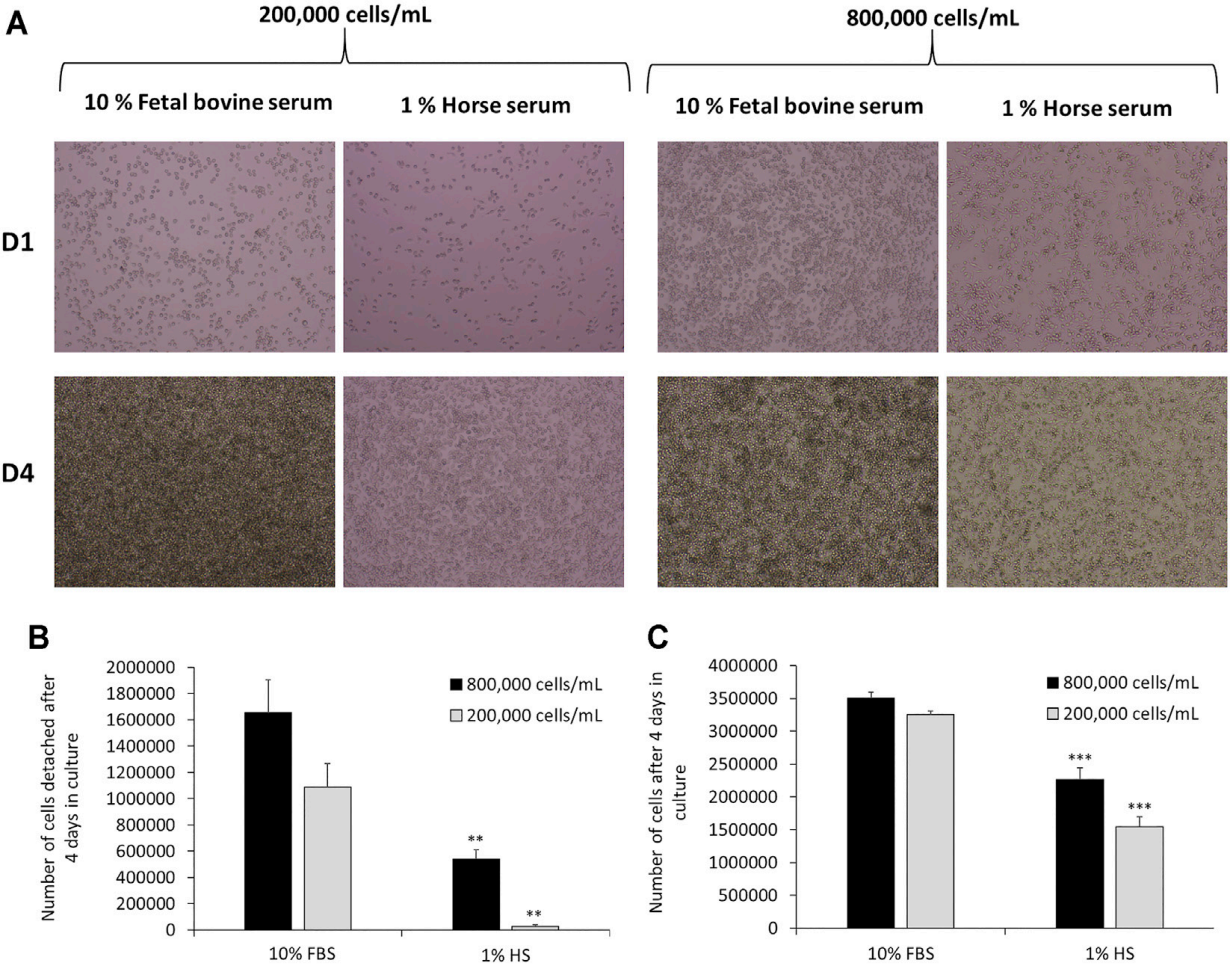
Comparison of cell culture medium for J774 cells after 4 days in culture medium. Panel A- Microscopic observation of J774 cells after 1 day (D1) or 4 days (D4) in culture medium. Cell culture medium tested: DMEM +10% Fetal Bovine Serum (FBS) and DMEM +1% Horse Serum (HS). The seeding tested was 200,000 cells/mL or 800,000 cells/mL. Panel B- Quantification of detached cells in the supernatant *via* cell numeration. Quantification of adherent cells *via* crystal violet (N = 3). *, significant difference between the 10% fetal bovine serum and 1% horse serum conditions. Number of symbols: 2: *p* < 0,01, 3: *p* < 0.001.

J774A.1 cells were cultivated for 1 week in DMEM +10% FBS or DMEM +1% HS and the culture media were changed every 2 days. Cells were observed *via* optical microscopy after 4 days of culture (seeding of cells = day 0). Then, cells were quantified in order to determine the number of detached and adherent cells. After 4 days of culture, the results showed (Figure 2A) a strong slowdown of the multiplication of J774 in DMEM supplemented with 1% HS in comparison to 10% FBS. This applies to both high (800,000 cells/ml) and low seeding concentrations (200,000 cells/ml). In DMEM +1% HS, microscopic observation revealed that cells were less round and more stretched (with filamentous extensions). In the collected culture media, the quantification of cells (Figure 2B) revealed much less detached cells in 1% HS (−68% with a seeding of 800,000 cells/ml and −63% with a seeding of 200,000 cells/ml, compared to the cells cultivated in DMEM +10% FBS). Moreover, the quantification of adherent cells revealed a significant decrease of 35 and 52% in comparison to 10% FBS for a seeding of 800,000 cells/ml and 200,000 cells/ml respectively (Figure 2C, thereby confirming a lower cell proliferation in 1% HS compared to 10% FBS.

#### Assessment of Cell Viability in Culture Medium DMEM +1% Horse Serum

**FIGURE 3:**
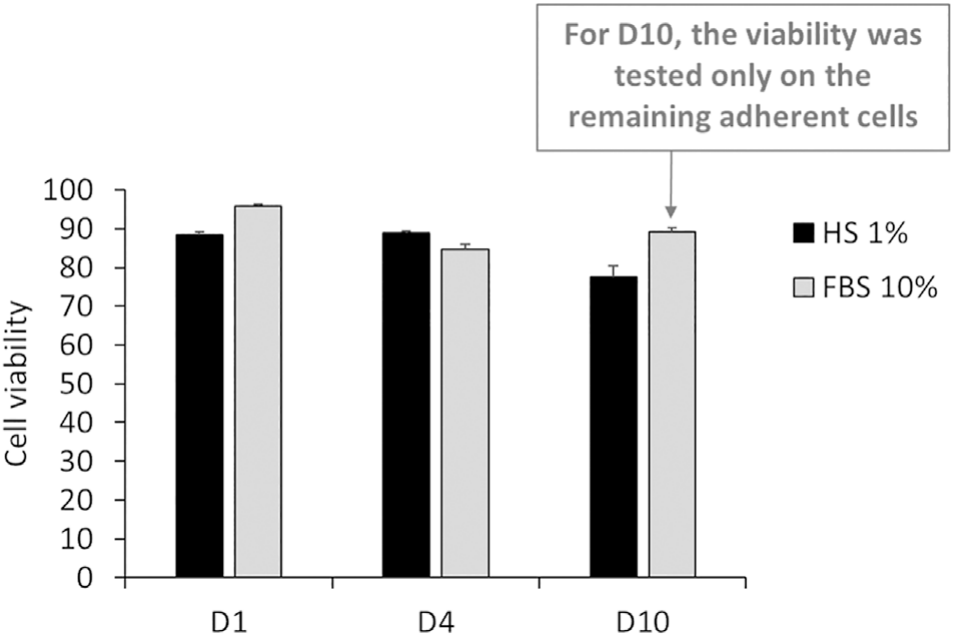
Percentage of living cells after 24 h (D1), 4 days (D4) and 10 days (D10) of culture in DMEM +1% Horse Serum (1% HS). Black bars; culture medium supplemented with DMEM +1% Horse Serum (1% HS). Grey bars; culture medium supplemented with DMEM +10% Fetal Bovine Serum (FBS) (N = 3).

After 24 h of culture in DMEM +1% HS, the viability of J774 cells was similar to the one in 10% FBS. Indeed, the results in Figure 3 showed that only 11.6% of cells died (viability = 88.6%), whereas the condition with 10% FBS showed a cell death of 4.25% (Viability = 95.75%). However, this rate can be variable according to experiments. Typically, cell viability in DMEM +10% FBS, was about 90% (Torres et al., 2020a). After 4 days of culture in DMEM +1% HS, viability was 88.8% and after 10 days, viability decreased slightly to reach 77.6%. In comparison, in DMEM +10% FBS, viability was 84.68%. and increased to 89% for 10% FBS after 4 days. However, for the DMEM+10% FBS condition, many more cells were detached. Thus, many cells were eliminated of the experiment after each renewal of the culture medium. Consequently, this viability level is not relevant.

#### Comparison of Culture Conditions: Maintaining of the Main Functionalities of Macrophages (Phagocytic and Inflammatory Capacities)

**FIGURE 4:**
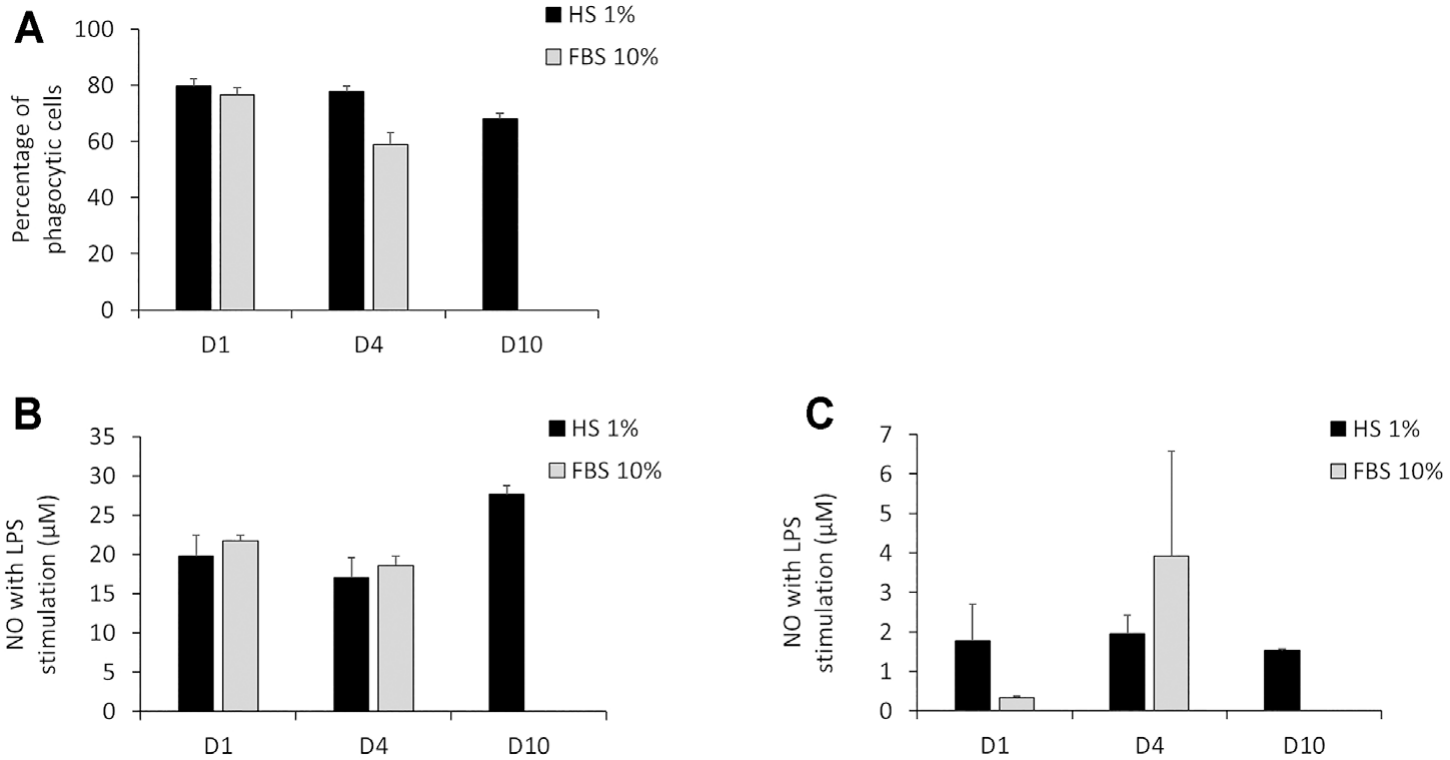
Main functionalities measurement of macrophages. **(A)** Percentage of phagocytic cells measured *via* engulfment of Y/G fluorescent latex beads. **(B)** NO secretion with LPS stimulation for 12 h. **(C)** NO secretion without LPS stimulation. 24 h (D1), 4 days (D4) and 10 days (D10) of culture in DMEM +1% horse serum (1% HS). Black bars; culture medium supplemented with DMEM +1% Horse Serum (1% HS). Grey bars; culture medium supplemented with DMEM +10% Fetal Bovine Serum (FBS) (N = 3). No measurements were made for cells maintained in DMEM+10% FBS because of the important cell detachment occurring, as mentioned in the previous section.

The results in Figure 4A show that after 24 h of culture in DMEM +1% HS, the percentage of phagocytic cells was 79.7%. This percentage was similar after 4 days (77.6%). After 10 days of culture in DMEM +1% HS, the percentage of phagocytic cells decreased to 68%. Despite this slight reduction of the phagocytic capacity, after 10 days in culture medium, a great majority of cells were still able to maintain their phagocytic activity. While the initial phagocytosis was similar for DMEM +1% HS and DMEM +10% FBS, phagocytosis seemed to have a tendency to decrease in DMEM +10% FBS medium after 4 days in culture (only 59% of cells maintain their phagocytic capacity). For NO secretion, the results in Figure 4B show that in DMEM +1% HS, J774A.1 cells maintained an adequate response to LPS (100 ng/ml) stimuli for the final 18 h of culture. Indeed, the concentration of NO was 19.8 µM after 24 h of culture, 17 µM after 4 days of culture and 27.7 µM after 10 days of culture. Thus, cells appear to be more sensitive when cultured for a long time. Without LPS stimulation, NO secretion was very low: between 1.5 and 2 µM regardless of cell culture duration (Figure 4C), showing that culture conditions did not induce a spurious response at this level. In DMEM+10% FBS, results showed after 4 days of culture, the NO secretion seems less stable with a higher standard deviation.

**FIGURE 5:**
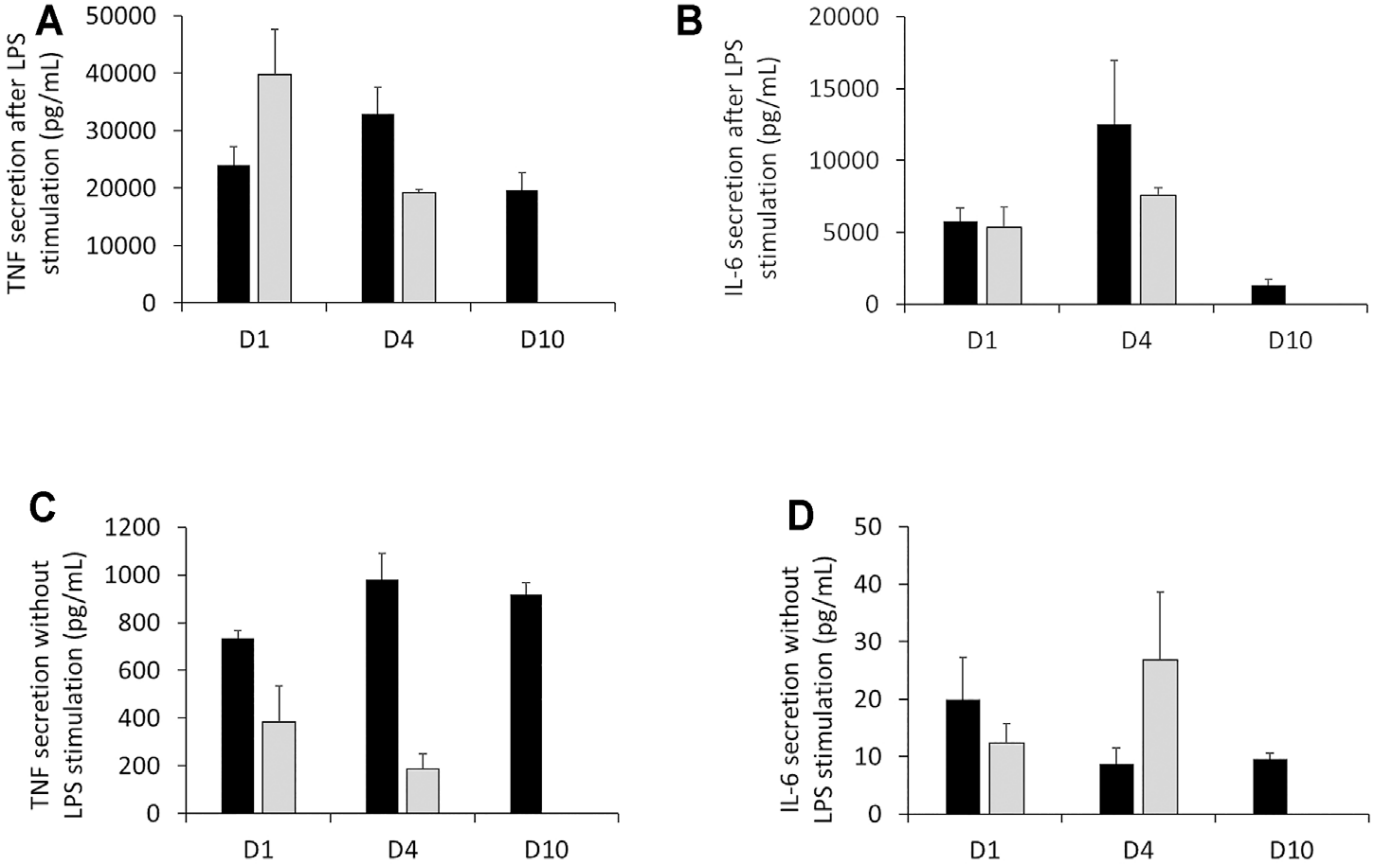
Measurement of TNF secretion and IL-6 cytokines in the presence or absence of LPS stimulation. **(A)** Assessment of TNF released by J77A.1 cells with LPS stimulation (pg/ml). **(B)** Assessment of the IL-6 released by J774A.1 cells with LPS stimulation (pg/ml). **(C)** Assessment of TNF released by J774A.1 cells without LPS stimulation (pg/ml). **(D)** Assessment of IL-6 released by J774A.1 cells without LPS stimulation (pg/ml) (N = 3). Black bars; culture medium supplemented with DMEM +1% Horse Serum (1% HS). Grey bars; culture medium supplemented with DMEM +10% Fetal Bovine Serum (FBS). No measurements were made for cells maintained in DMEM+10% FBS at day 10, because of the important cell detachment, as mentioned in the previous section.

We chose to extend our assessment of the inflammatory ability according to the duration of culture in DMEM supplemented with 1% HS. Regarding TNF and IL-6, the results in Figure 5 showed that J774A.1 had a strong response to LPS (100 ng/ml) stimulation for short and long-term cell culture in DMEM +1% HS. Indeed, despite a variability of responses (especially for IL-6) dependent on duration of culture, for the three conditions, the secretion of TNF and IL-6 was significantly higher than in the conditions without LPS stimulation. For example, with LPS stimulation, TNF secretion was comprised between 19,604 and 32,935 pg/ml (Figure 5A) and IL-6 secretion was between 1,350 and 12,547 pg/ml (with high standard deviations) (Figure 5B). Without LPS, TNF secretion was comprised between 736 and 981 pg/ml Figure 5C) and IL-6 secretion was between 20 pg/ml (with large standard deviations) and 8.7 pg/ml according to duration of cell culture (Figure 5D). In comparison to DMEM supplemented with 10% FBS, even if the raw values were different, the results showed that the orders of magnitude of TNF and IL-6 concentrations were the same for the two culture conditions, both in the presence and absence of LPS. That is, in the presence of LPS, TNF secretion is between 39,777 and 19,175 pg/ml, and for IL-6, it is between 5,346 and 7,579 pg/ml. In the absence of LPS, TNF secretion drops to 383 and 187 pg/ml while IL-6 concentration ranges from 12.3 to 26.8 pg/ml.

As mentioned in *Assessment of cell viability in culture medium DMEM + 1% horse serum* above, strong detachment was observed for cells cultured for 10 days in DMEM+10% FBS. This meant that the cultures were no longer exploitable, so that no functional measurements were performed for this precise condition.

### Validation of the DMEM +1% HS Culture Medium System for Medium-Term (4 days) Experiments: Example of the Effects of Silica-Based Pigments

**FIGURE 6:**
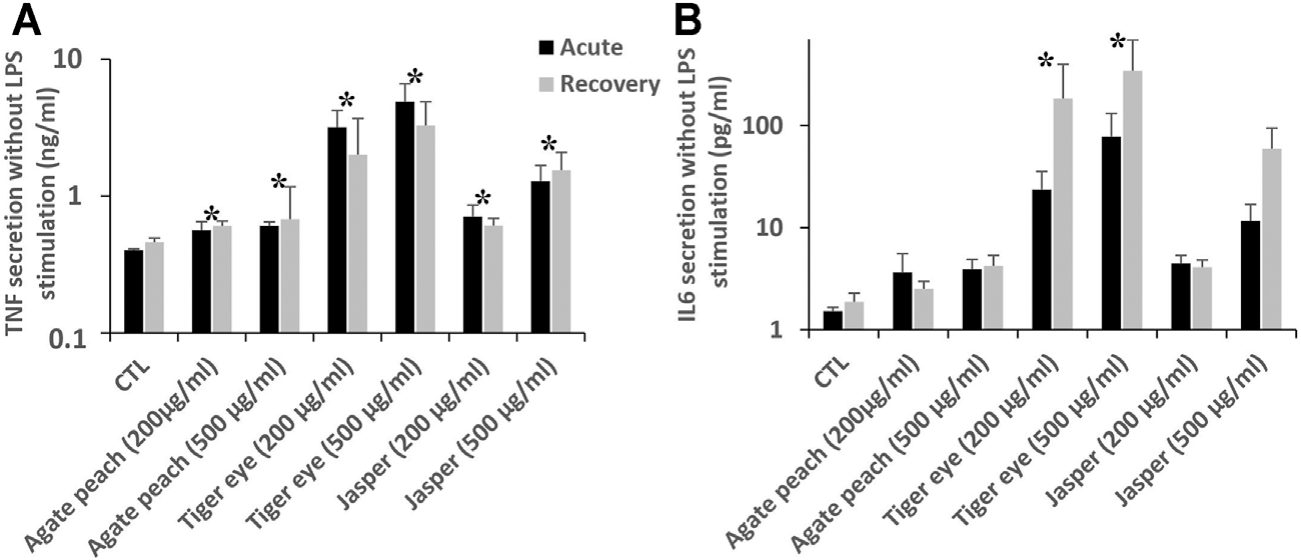
Intrinsic cytokine secretion in J774A.1 cells after exposure to pigment containing silica, followed or not by a recovery period of 72 h. Black bars for acute exposure and grey bars for recovery protocol. Data are represented as Mean +SD **(A)** TNF secretion **(B)**. IL-6 secretion All the treated cells are significantly different from the secretion of the control (Mann-Whitney statistic test, *p* < 0.05) in both acutely exposed cells and recovery scenario (N = 4).

The characterization of the silica-based pigments was carried out by transmission electronic microscopy and is shown in Supplementary Figure S1. It confirms a high variety of shapes and sizes of the tested particles with micrometric particles, as expected from the supplier specifications, but also sub-micrometric particles. Working doses of 200 and 500 μg/ml, corresponding respectively to one and 2.5 of particles (5 µm nominal diameter) per cell, were used. These concentrations proved to be non-cytotoxic during the timespan of the experiments (4 days). In these experiments, the parameter tested was cytokine release, as various forms of silica are known to induce inflammatory responses *in vivo* (Arts et al., 2007) and *in vitro* (e.g., in (Wiemann et al., 2016; Torres et al., 2020a)). The results, shown in Figure 6, revealed a secretion of pro-inflammatory cytokines after exposure to these silica-based pigments.

First, for tumor necrosis factor α (TNF), all the pigment-exposed cells showed a significantly higher TNF secretion. In the control cells, a basal TNF secretion of 400 pg/ml was observed. After an acute exposure to pigments, TNF secretion was increased from 560 pg/ml (agate) to 4,800 pg/ml (tiger eye 500 μg/ml). These increases were largely persistent even after the 72 h recovery period. Then, after an acute exposure there is a significant increase of IL-6 secretion, which become detectable (24 and 78 pg/ml), for cells exposed to tiger eye. After the recovery period, the cells exposed to tiger eye are still secreting IL-6 in higher doses than just after the exposure (185 and 346 pg/ml). There is therefore a persistence and a delayed effect after exposure to tiger eye. After the recovery period, there is also a significant detectable signal of IL-6 secretion (60 pg/ml) after the exposure to jasper at the higher dose (500 μg/ml). Here again, a dose dependency phenomenon could be observed.

### Validation of the DMEM +1% HS Culture Medium System for Long-Term (10 days) Experiments: Example of the Effects of a Cobalt-Based Pigment

The characterization of the cobalt-based pigment PB28 (cobalt blue) was carried out by transmission electronic microscopy and is shown in Supplementary Figure S1. Here again, micron-sized and sub-micron sized particles were observed. Preliminary experiments led to determine that the LD 20 was reached at 500 μg/ml. Corundum was used as a negative control (Wiemann et al., 2016) at the same concentration.

#### Phagocytic Capacity

**FIGURE 7:**
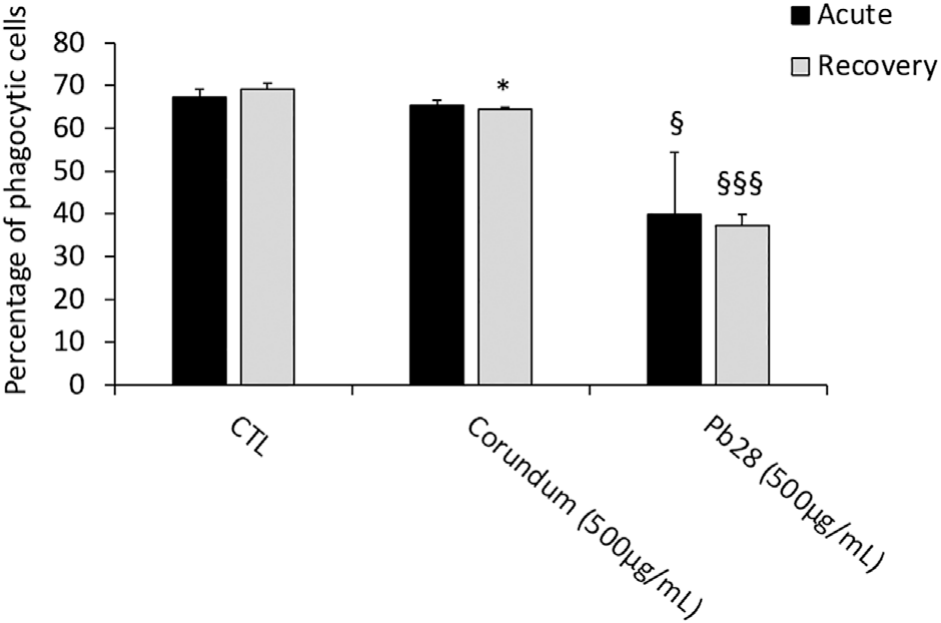
Phagocytic capacity. Black bars; acute exposure (last day of culture = 24 h). Grey bars; recovery exposure (first day of culture = 24 h) and 9 days recovery post exposure. Percentage of cells able to phagocyte fluorescent Y/G labeled latex beads. Data are represented as Mean +SD. Significance symbols: *, significant change compared to negative control only (cells without treatment). §, significant change compared to negative control and corundum control (same concentration). Number of symbols: 1: *p* < 0.05, 2: *p* < 0.01, 3: *p* < 0.001 (Student *t* test, N = 3).

Results in Figure 7 showed that for PB28 (500 μg/ml), in acute exposure mode, the percentage of phagocytic cells dropped dramatically (about 40%) when compared to controls. Interestingly enough, after the 10 days post-exposure recovery, the results showed that the proportion of phagocytic cells remained very low, as in acute exposure mode (−47% and −42% when compared to control without treatment and corundum (500 μg/ml). Regarding the corundum control, a slight decrease was observed after recovery, suggesting a slight effect due to a high concentration of particles regardless of the material used. However, we could conclude that PB28 (500 μg/ml) has a significant, long-lasting effect on the phagocytic capacity of J774A.1 macrophages.

#### Inflammatory Ability (Nitric Oxide and Pro-inflammatory Cytokine Secretion)

In these experiments, we assessed the intrinsic effects of particles after 10 days of culture, in acute and recovery exposure modes, after a challenge with PB28 or corundum.

##### NO Secretion

**FIGURE 8:**
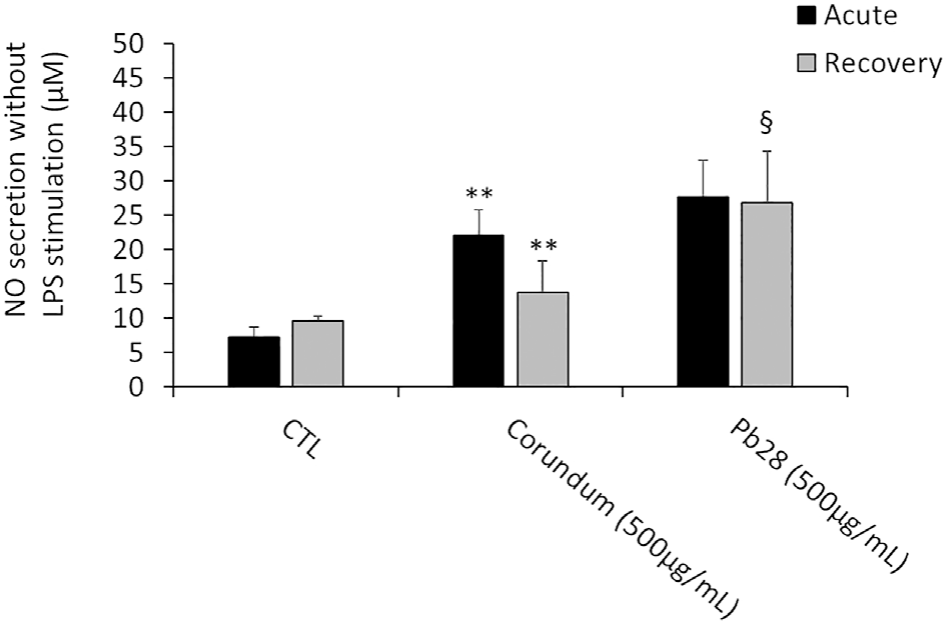
NO secretion. Black bars; acute exposure (last day of culture = 24 h). Grey bars; recovery exposure (first day of culture = 24 h) and 9 days recovery post exposure. Data are represented as Mean +SD. Significance symbols: *, significant change compared to negative control only (cells without treatment). §, significant change compared to negative control and corundum control (same concentration). Number of symbols: 1: *p* < 0.05, 2: *p* < 0.01 (Student *t* test, N = 3).

Without LPS stimulation and under acute exposure, the results (Figure 8) showed a significant increase of NO secretion for corundum-treated cells (500 μg/ml) in comparison to the negative control. For PB28-treated cells (500 μg/ml), the NO production appeared to be similar to that of corundum-treated cells. Thus, the results suggested that the internalization of a high quantity of particles had a pro-inflammatory effect for these two chemically different particles.

After 10 days post-exposure recovery (Figure 8), the concentration of NO released after exposure to corundum returned to values that were not significantly different from the negative controls. However, a persisting increase of NO secretion was observed for PB28-treated cells in comparison to negative controls and to corundum-treated cells.

##### IL-6 and TNF Secretion

**FIGURE 9:**
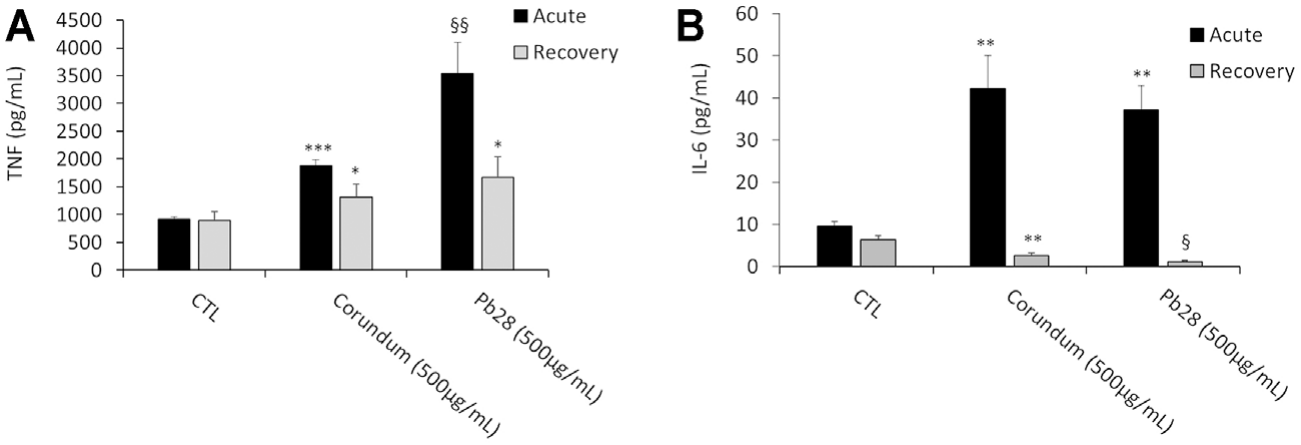
Pro-inflammatory cytokines secretion. Black bars; acute exposure (last day of culture = 24 h). Grey bars; recovery exposure (first day of culture = 24 h) and 9 days recovery post exposure. Data are represented as Mean +SD. Panel A- TNF secretion without LPS stimulation. Panel B- IL-6 secretion without LPS stimulation. Significance symbols: *, significant change compared to negative control only (cells without treatment). §, significant change compared to negative control and corundum control (same concentration). Number of symbols: 1: *p* < 0.05, 2: *p* < 0.01, 3: *p* < 0.001 (Student *t* test, N = 3).

In these experiments, only the intrinsic effects of corundum and cobalt blue were investigated. In the acute exposure mode, the results in Figure 9B showed a significant increase of IL-6 secretion for corundum (500 μg/ml) and PB28 (500 μg/ml) in comparison to control without treatment. However, despite this increase, no significant difference was observed between corundum and PB28 However, regarding TNF secretion (Figure 9A), even if the results also showed an increase of TNF for corundum and PB28, the TNF secretion in the presence of PB28 (3542,5 pg/ml) was significantly higher than in the presence of corundum (1878,4 pg/ml) while the basal TNF secretion in the control was 916.9 pg/ml.

In the recovery exposure mode (Figure 9A), the TNF secretion after 9 days of recovery was maintained at 1,308.6 and 1,677.1 pg/ml for corundum and PB28, respectively, when compared to negative control (896.4 pg/ml). Thus, regarding TNF, a persisting inflammatory effect was observed even if the values are lower than in acute exposure mode. Surprisingly enough, for IL-6 (Figure 9B), the concentration decreased in the presence of corundum (2.6 pg/ml) and for PB28, the concentration declined drastically (1.1 pg/ml). The results suggest persisting inflammation disorders in the presence of a high quantity of particles. This phenomenon is exacerbated in the presence of cobalt.

## Discussion

A critical aspect in *in vitro* toxicology is to use conditions that are as close as possible as those prevailing *in vivo*. This aspect is even more important for toxicological studies that investigate responses on a longer time scale than the few hours usually used in most *in vitro* toxicological studies. In this respect, the proliferation dimension is a key aspect to be taken into account in such medium/long term *in vitro* studies. Thus, for epithelial cells that proliferate at a relatively high rate *in vivo*, the proliferation must be kept in the *in vitro* systems. In the frame of toxicological studies, this has been the case for keratinocytes (Comfort et al., 2014), lung (Gliga et al., 2018; Murugadoss et al., 2021) or intestinal (Chen et al., 2016; Vila et al., 2017; Murugadoss et al., 2021) epithelial cells.

For maintaining cell proliferation fetal bovine serum is the additive of choice in cell culture. It has long been shown to be the most active serum for inducing cell proliferation in a wide variety of systems (Puck et al., 1958; Sirbasku and Kirkland, 1976; Yasin et al., 1981; Zamansky et al., 1983). Its widespread use is thus legitimate for such proliferating cells.

The situation is quite different for immune cells. In the immune system, only the immature cells proliferate, and the mature immune cells are post-mitotic. The only exceptions known are the clonal expansion of stimulated lymphocytes (Williamson et al., 1976; Lee et al., 2002), and the slow self-renewal of resident macrophages (Hashimoto et al., 2013; Ginhoux and Jung, 2014; Ginhoux and Guilliams, 2016). When using cell lines representing immune cells, the proliferation induced by fetal bovine serum becomes a problem in medium/long-term experiments.

Because of their key role in the control of the inflammatory responses, macrophages are indeed an important cell type to be tested in toxicology, and especially in the toxicology of persistent materials and chemicals. There are thus numerous publications that use *in vitro* models of macrophages to test immunotoxicity *sensu lato*, i.e., including inflammatory responses. However, most of these publications use a short-term exposure, and are thus unable to address the question of persistence. In the few papers that do address this question (Dalzon et al., 2019b; Torres et al., 2020a), or that study the effects of repeated exposures (Dalzon et al., 2020a; Torres et al., 2020b; Murugadoss et al., 2021) technical problems linked to cell proliferation were encountered. This discrepancy between the classical cell growth in culture and the negligible macrophage proliferation were addressed by working at high cell concentrations (confluency) in some cases (Dalzon et al., 2019b, 2020a; Torres et al., 2020a, 2020b) and neglected in other studies (Murugadoss et al., 2021). There is thus a need for a system that would limit cell proliferation, while keeping cell viability and functionality for an extended period.

Serum-free culture has been advocated by several authors (Wiemann et al., 2016; Leibe et al., 2019). While it does inhibit proliferation, it also exacerbates cytotoxicity and the kinetics of the cellular responses (Leibe et al., 2019). While the use of serum-free cultures may be relevant for the study of pulmonary toxicity, although this can be considered as an open question due to the presence of serum proteins in the pulmonary surfactant (Wattiez et al., 2000), serum-free cultures cannot represent the conditions prevailing in the body. Furthermore, cell viability cannot be maintained for extended time periods in serum-free culture, which precludes the use of these systems for any study on effect persistence. There is thus a need for a system that would both decrease cell proliferation and keep cell viability and functionality for extended times.

Based on this reasoning, the use of horse serum, and especially of low concentrations thereof, is an already documented, albeit rarely used, process used to decrease proliferation and increase differentiation, as described in several systems (Yaffe and Dym, 1973; Auersperg, 1978; Fedoroff and Hall, 1979; Czarnetzki and Behrendt, 1981; Brunner and Tschank, 1982; Schlüns and Drewes, 1984; Voisin et al., 1993). These systems take advantage of the different growth support properties of fetal bovine serum and of adult horse serum (Yasin et al., 1981). They use fetal bovine serum to passage and amplify the cell lines of interest. Once the adequate density required for the experiments is reached, the cells are switched to a medium containing horse serum to stop proliferation and induce differentiation (Yaffe and Dym, 1973; Auersperg, 1978; Fedoroff and Hall, 1979). The system described in the present paper works exactly on the same principle. The J774A1 cells are propagated and expanded using classical culture conditions (10% fetal bovine serum). Once the adequate density has been reached, the culture medium is changed to a medium containing only 1% horse serum.

This use of low concentrations of horse serum did decrease proliferation, favored attachment to the support, which is also a critical feature to avoid spurious cell selection by detachment from the culture support, and helped in maintaining the differentiated functions (e.g; phagocytosis or cytokine production) of macrophages cell lines over time. Moreover, in the frame of the 3R approach, the use of low concentrations of horse serum is of higher ethical standards than the use of fetal bovine serum, as horse serum usually comes from donor herds, while fetal bovine serum comes from slaughterhouses.

The application examples showed that this culture system kept sensitivity not only to the dose but also to structure of the tested toxicants, as exemplified on the silica pigments. The culture system was able to reveal that the response did not only depend of the particle size or of the mineral, but also of its composition, form and surface. The three silica based pigments are indeed composed of silica colored by iron oxide. However, the fine structure of the three minerals differ, mostly in the fine arrangement of the quartz microcystallites in the minerals. In this comparative study, the tiger eye is the most inflammatory material. Tiger eye is composed of quartz crystals and fibers of amphibole, which is quite similar to asbestos composition (Janik and Wrona, 2017). Indeed, tiger eye is sometime considered as a variety of amosite, which is one form of asbestos. The important point of these experiments is that this simple *in vitro* system allowed demonstrating the persistence of the inflammatory effects when challenged with well-known persistent proinflammatory substances.

The second application example on the cobalt pigment showed that the positive features of the culture system could be used on a material with completely different characteristics (e.g., different chemical composition), and that the persistence of the effects could be studied on a longer time scale (at least up to 10 days) and on a variety of parameters, so that the system is not restricted to cytokine release studies.

In conclusion, the low serum culture system described here represents an easy to implement system that allows performing toxicological studies on macrophages for extended time periods, using widely-available cell lines. Such a system should increase the type and relevance of *in vitro* immunotoxicological studies.

## Data Availability

The raw data supporting the conclusions of this article will be made available by the authors, without undue reservation.
