# Supplementary material for: A Low-Serum Culture System for Prolonged in Vitro Toxicology Experiments on a Macrophage System
Source: Front Toxicol. 2021 Dec 6;3:780778. doi: 10.3389/ftox.2021.780778 (PMC8915817; doi:10.3389/ftox.2021.780778)
Supplement: Supplementary file 1 [file Image1.pdf]

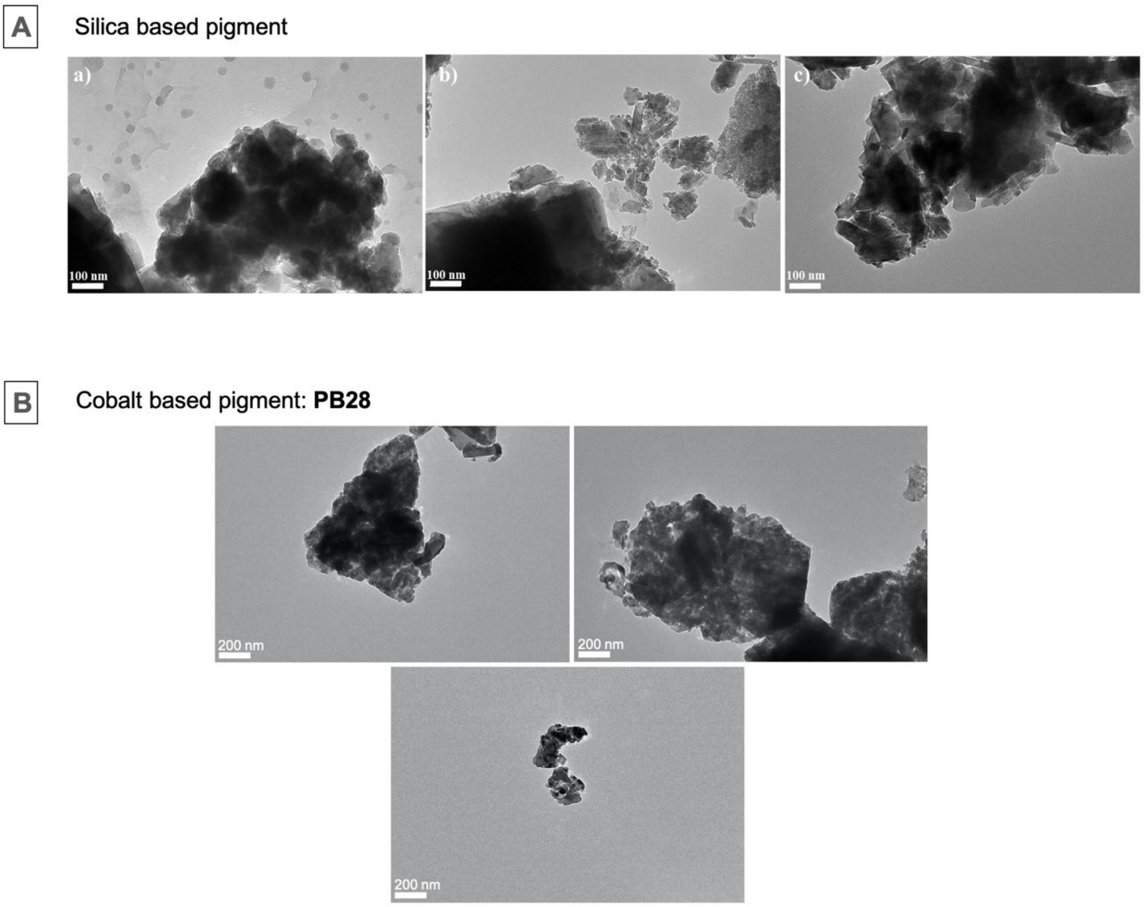

Figure S1: Transmission microscopy electronic (TEM). A- Silica based particles composing pigments: a) Agate peach (quartz), b) Tiger eye (crystal of quartz, fibre, iron), c) Jasper (80% of quartz). Scale bare = 100nm. B- different TEM images of Cobalt based particle composing pigments (named PB28). Scale bar = 200nm.
